# Validation and quality measurements for STS, EuroSCORE II and a regional risk model in Brazilian patients

**DOI:** 10.1371/journal.pone.0238737

**Published:** 2020-09-10

**Authors:** Omar Asdrúbal Vilca Mejia, Gabrielle Barbosa Borgomoni, Jorge Passamani Zubelli, Luís Roberto Palma Dallan, Pablo Maria Alberto Pomerantzeff, Marco Antonio Praça Oliveira, Orlando Petrucci Junior, Marcos Gradim Tiveron, Marcelo Arruda Nakazone, Rafael Ângelo Tineli, Valquíria Pelisser Campagnucci, Roberto Rocha e Silva, Alfredo José Rodrigues, Walter José Gomes, Luiz Augusto Ferreira Lisboa, Fábio Biscegli Jatene, REPLICCAR Study Group

**Affiliations:** 1 Department of Thoracic and Cardiovascular Surgery, Heart Institute, University of São Paulo Medical Center, São Paulo, São Paulo, Brazil; 2 Department of Cardiovascular Surgery, Hospital Samaritano Paulista, São Paulo, São Paulo, Brazil; 3 National Institute for Pure and Applied Mathematics, Rio de Janeiro, Rio de Janeiro, Brazil; 4 Team of Prof. Dr. Sérgio Almeida de Oliveira in Hospital Real and Benemérita Sociedade Portuguesa de Beneficência, São Paulo, São Paulo, Brazil; 5 Department of Surgery, Faculty of Medical Science, State University of Campinas, Campinas, São Paulo, Brazil; 6 Department of Surgery, Irmandade Santa Casa de Marília, Marília, São Paulo, Brazil; 7 Department of Cardiology and Cardiovascular Surgery, Hospital de Base de São José do Rio Preto, São José do Rio Preto, São Paulo, Brazil; 8 Department of Surgery, Irmandade Santa Casa de Piracicaba, Piracicaba, São Paulo, Brazil; 9 Department of Surgery, Irmandade Santa Casa de São Paulo, São Paulo, Brazil; 10 Department of Surgery, Paulo Sacramento Hospital, Jundiaí, São Paulo, Brazil; 11 Cardiovascular and Thoracic Surgery Division at Ribeirão Preto Medical School - USP, Ribeirão Preto, São Paulo, Brazil; 12 Cardiovascular Surgery Department of the Federal University of São Paulo - Escola Paulista de Medicina, São Paulo, São Paulo, Brazil; Case Western Reserve University School of Medicine, UNITED STATES

## Abstract

**Objectives:**

The objectives of this study were to describe a novel statewide registry for cardiac surgery in Brazil (REPLICCAR), to compare a regional risk model (SPScore) with EuroSCORE II and STS, and to understand where quality improvement and safety initiatives can be implemented.

**Methods:**

A total of 11 sites in the state of São Paulo, Brazil, formed an online registry platform to capture information on risk factors and outcomes after cardiac surgery procedures for all consecutive patients. EuroSCORE II and STS values were calculated for each patient. An SPScore model was designed and compared with EuroSCORE II and STS to predict 30-day outcomes: death, reoperation, readmission, and any morbidity.

**Results:**

A total of 5222 patients were enrolled in this study between November 2013 and December 2017. The observed 30-day mortality rate was 7.6%. Most patients were older, overweight, and classified as New York Heart Association (NYHA) functional class III; 14.5% of the patient population had a positive diagnosis of rheumatic heart disease, 10.9% had insulin-dependent diabetes, and 19 individuals had a positive diagnosis of Chagas disease. When evaluating the prediction performance, we found that SPScore outperformed EuroSCORE II and STS in the prediction of mortality (0.90 vs. 0.76 and 0.77), reoperation (0.84 vs. 0.60 and 0.56), readmission (0.84 vs. 0.55 and 0.51), and any morbidity (0.80 vs. 0.65 and 0.64), respectively (*p*<0.001).

**Conclusions:**

The REPLICCAR registry might stimulate the creation of other cardiac surgery registries in developing countries, ultimately improving the regional quality of care provided to patients.

## Introduction

Multicenter registries in cardiac surgery constitute the basis for most of the progress achieved in the United States and the European Union [1, 2]. However, developing countries have been slow to join the quality improvement (QI) movement, perhaps because of differences in socio-demographic and healthcare characteristics [3].

In a state where up to 80% of all cardiac surgery procedures are reimbursed by the federal Unified Health System (SUS), development of regional data collection mechanisms for QI and safety is essential to serve as a basis for clinical guidelines and healthcare policies. Of importance, increasing international literature with regional data allows researchers and policy makers to fine-tune guidelines and policies to better align clinical practice with regional clinical and socioeconomic reality.

EuroSCORE II was published in 2012 [4] and was constructed with a dataset that includes over 20000 patients from 43 countries worldwide, mostly in Europe. This revised model was criticized for underestimating mortality from cardiac surgery compared to observed mortality. This raised concerns by suggesting to readers not to use it for scientific purposes or quality control [5]. However, validation studies have shown contradictory results; the only paper published in Brazil showed a failure in calibration in a single-center study [6].

The São Paulo Cardiovascular Surgery Registry (REPLICCAR) was created in 2013 with the goal of improving patient safety and implementing quality improvements in the São Paulo state network [7]. Funnel plots with 99% confidence intervals (CI) to assess risk-adjusted mortality compared to mean baseline mortality were constructed. Mortality did not differ from the administrative cardiovascular surgery database of the state of São Paulo [8].

Regarding the quality issues faced by developing countries for cardiac surgery procedures, the objective of this article is threefold: (1) to describe the REPLICCAR statewide registry, (2) to create the SPScore model for validation and comparison with EuroSCORE II and STS, and (3) to describe a novel web application that allows research peers, providers, policy makers, and patients to dynamically and directly explore REPLICCAR data to establish QI and safety initiatives.

## Materials and methods

### Overall description

#### Registry motivation

The REPLICCAR registry was funded through a partnership between the Secretary of Health of the State of São Paulo and the São Paulo Research Foundation (FAPESP).

#### Study design

REPLICCAR is a mandatory prospective registry currently involving 11 different centers around the state of São Paulo, Brazil. REPLICCAR included all consecutive adult patients undergoing cardiac surgical procedures involving Coronary artery bypass grafting (CABG) and/or heart valve surgery. No patients who met these criteria were excluded. The EuroSCORE II and STS variables were also prospectively collected for each patient, in each center, and the calculation was performed by researchers trained for this purpose at the coordinating center. SPScore was constructed so that it could be incorporated into regional data collection and management systems. To this end, REPLICCAR was divided into a developmental data set and a validation data set using random sampling from a binomial distribution. In the validation data, the performance of SPScore was compared with the performance of EuroSCORE II and STS. This study is described according to the applicable components of the SQUIRE statement [9].

### Ethics

The REPLICCAR project (SDC: 3853/12/109) was approved by the Research Ethics Committee of the University of São Paulo, Heart Institute, Hospital das Clínicas, University of São Paulo Medical School, Brazil. The Brazilian Platform for Health Research and the Institutional Review Board of each of the 11 currently participating sites approved the data collection for REPLICCAR registry.

### Setting

Data were collected at 11 different centers across the state of São Paulo, Brazil. These sites included the Beneficência Portuguesa de São Paulo, Santa Casa de Marília, Heart Institute (InCor) at the University of São Paulo, Santa Casa de São Paulo, the State University of Campinas, São José do Rio Preto Hospital, Santa Casa de Piracicaba, São Paulo Federal University, SOBAM Group, Hospital das Clínicas of the University of São Paulo at Ribeirão Preto and the Paulo Sacramento Hospital.

REPLICCAR participants were recruited from locations within the São Paulo Health Technology Assessment Network (HTA-NATSs/SES-SP). Perioperative patient data, including observed mortality data and follow-up until 30 days after surgery were collected on an online platform (http://bdcardio.incor.usp.br/). Data quality checks were performed on a regular basis, with feedback provided to individual centers when necessary.

The recruitment of participants for this study occurred between November 2013 and December 2017.

### Dynamic graphics for interaction with REPLICCAR data

We created a web application that allows dynamic data exploration to provide research peers, healthcare professionals, policy makers, and the general public with the ability to directly explore REPLICCAR data.

### SPScore model for quality improvement and safety initiative experiments

We developed a SPScore model (http://repliccar.incor.usp.br:3838/prediction/) and a subsequent web application (http://repliccar.incor.usp.br:3838/exploratory/) to enable the network of participants to experience QI and safety initiatives to reduce morbidity and mortality after cardiac surgery in the state of São Paulo.

### Data collection

A full data dictionary for the REPLICCAR registry in its current version is provided on the website www.repliccar.com.br. The following variables were considered for the SPScore model: planned procedure, age (calculated through date of birth), education, gender, body mass index (weight in kilograms divided by height squared in meters), previous myocardial infarction, time since infarction (in days), previous coronary stent, time from stent implantation to surgery, previous heart surgery, NYHA functional class, rheumatic heart disease, glycated hemoglobin levels, hematocrit, atrial fibrillation, ejection fraction, pulmonary artery systolic pressure, walking speed, insulin-dependent diabetes, creatinine clearance levels, urgency/emergency admission, presence of Chagas disease, isolated coronary artery bypass graft surgery, types of arterial grafts, presence of left ventricular aneurysm, procedures for mechanical complications, replacement or repair of aortic/mitral/tricuspid and/or pulmonary heart valve, concomitant ascending aortic procedure, and level of blood cell transfusion.

In-hospital death was defined as death during hospital stay or up to 30 days after surgery if hospital discharge occurs. In addition, the other outcome variables up to 30 days after surgery included: (1) reoperation, defined as any type of reoperation, including reoperation for bleeding; (2) readmission within 30 days after surgery; (3) any morbidity, defined as the presence of cardiac complications (presence of postoperative ventricular arrhythmia, atrial fibrillation, myocardial infarction or cardiogenic shock), acute renal failure, any infection (presence of acute endocarditis, wound infection, mediastinitis, pneumonia, or sepsis during hospitalization), and other complications, including systemic inflammatory response syndrome and stroke.

### Modeling strategy

Our exploratory analysis started by evaluating distributions, frequencies, and percentages for each of the continuous and categorical variables. Categorical variables were evaluated for near-zero variation [10]. Extensive graphical displays were used for both univariate analysis and bivariate associations, accompanied by broader tests, such as maximal information coefficient [11] and nonnegative matrix factorization [12] algorithms for continuous variables. The missing data were explored using a combination of graphical displays involving univariate, bivariate, and multivariate methods.

We modeled outcomes and predictors in the format described in the previous sections. To create a good predictive model, we evaluated a series of different machine learning classification models for predicting categorical variables, including Neural Network, Boosting, Multivariate Adaptive Regression Splines, Generalized Linear Model, Bagging, Nearest Neighbors, Support Vector Machines, Random Forest, Decision Tree, Linear Discriminant Analysis, Penalized Discriminant Analysis, Shrinkage Discriminant Analysis, Naive Bayes and Generalized Partial Least Squares. The validation followed a nested resampling strategy, in which the outer resampling is used to reduce overfitting, adopting a 5-fold cross-validation; the inner resampling was used for hyperparameter tuning using a 3-fold cross-validation strategy.

In situations where the outcome measures were imbalanced and, therefore, led the models to overestimate the class more often, we used the Synthetic Minority Over-sampling Technique (SMOTE). SMOTE creates synthetic data points for the minority class using a k-nearest neighbor approach. The number of synthetic data points was limited to avoid overestimating the minority class. Comparisons across models were performed using metrics for the area under the curve. The area under the curve ranges from 0 to 1, with 1 corresponding to perfect accuracy and 0.5 corresponding to random chance. The final model, named SPScore, was chosen based on a simultaneous combination of clinical face validity (reflecting current knowledge in the field of cardiac surgery) and predictive accuracy (maintaining the area under the ROC curve at 80% or more). Our modeling strategy involved the comparison only the test data, as multiple comparisons would increase the odds of model overfitting. For STS and EuroSCORE II models, we used simple Generalized Linear Models. The performance of the SPScore model was then compared to EuroSCORE II and STS in the validation data set for plotting calibration curves (comparing the observed and predicted mortality) and for discrimination (using the area under the ROC curve).

Calibration plots were constructed using Friedman’s super-smoother methodology on ungrouped data, while displaying the observed versus expected mortality trend [13]. Based on the ranked predicted risk, we evenly split our data cohort into 10 equally sized groups. Funnel plots were constructed for risk-adjusted mortality, using SPScore, EuroSCORE II, and STS as a reference for the expected data, following the Spiegelhalter methodology [14], using as criteria the 99% CI (reference mortality was the overall mortality in the 2013–2016 administrative cardiovascular surgery database of the state of São Paulo [10]) and sample size as a precision parameter.

## Results

Eleven Brazilian centers participated in the study. The final REPLICCAR registry contained records from 5222 patients. Data of the prevalence of risk factors and predictive variables are shown in Table 1. The mean patient age was 60.6 years. Most patients were in their late 50s and early 60s, overweight, and 10.9% of them had insulin-dependent diabetes. Approximately 43% were classified as NYHA functional class III and IV, the average EuroSCORE II was 3.1, and the STS was 1.0. Rheumatic heart disease was present in 14.5% of all patients, and 19 individuals had a positive diagnosis of Chagas disease (Table 1).

**Table 1:** Sample characteristics for the REPLICCAR registry.

Variable	Total (5222) n (%)
**Age at surgery**	60.6 (12) ^a^
**Female gender**	1900 (36.4%)
**BMI**	26.8 (4.58) ^a^
**NYHA functional class**	
I	1031 (19.7)
II	1944 (37.2)
III	1917 (36.7)
IV	330 (6.32)
**Insulin-dependent diabetes**	571 (10.9%)
**Walking speed test**	0.17 (0.62) ^a^
**Previous myocardial infarction**	1540 (29.5%)
**Rheumatic heart disease**	758 (14.5%)
**Glycated hemoglobin**	2.39 (3.48) ^a^
**Positive for Chagas disease**	19 (0.36%)
**EuroSCORE II**	3.1 (5.53) ^a^
**STS**	1.0 (1.06) ^a^
**Previous coronary stent**	548 (10.5%)
**Ejection fraction**	58.1 (11) ^a^
**Creatinine clearance**	73.6 (28.5) ^a^

BMI: Body mass index.

^a^ Median (Standard Deviation).

Table 2 summarizes the number of patients per type of procedure and the classification as elective, urgent, or emergency surgical procedures. Concerning the registry’s 30-day outcomes, atrial fibrillation was the most common complication (9.75%), followed by wound infection and pneumonia. Mortality rate was 7.64% and reoperations occurred in 4.19% of the patients (Table 3).

**Table 2:** Types of procedures performed.

Procedures performed	Total (5222) n (%)
**Surgery type**	
Elective	3.144 (59.6)
Urgent	2,039 (39)
Emergency	69 (1.32)
**CABG**	3146 (60.2)
**Bioprosthetic aortic valve replacement**	1002 (19.2)
**Bioprosthetic mitral valve replacement**	712 (13.6)
**Mitral valve repair**	351 (6.72)
**Mechanical mitral valve replacement**	215 (4.12)
**Mechanical aortic valve replacement**	163 (3.12)
**Tricuspid valve repair**	143 (2.74)
**Aortic valve repair**	59 (1.13)
**Ascending aortic surgery**	43 (0.82)
**Bioprosthetic tricuspid valve replacement**	11 (0.21)
**Mechanical tricuspid valve replacement**	3 (0.06)
**Heart valve surgery + CABG**	285 (5.46)
**AVRR + CABG**	189 (3.62)
**MVRR + CABG**	116 (2.22)
**AVRR + MVRR**	240 (4.6)

AVRR: aortic valve replacement or repair; MVRR: Mitral valve replacement or repair.

**Table 3:** Study outcomes.

Variable	Total (5222) n (%)
**Readmission**	133 (2.55)
**Death**	399 (7.64)
**Reoperation**	219 (4.19)
**Atrial fibrillation**	509 (9.75)
**Ventricular arrhythmia**	176 (3.37)
**Myocardial infarction**	64 (1.23)
**Cardiogenic shock**	241 (4.62)
**Renal failure**	405 (7.76)
**Endocarditis**	84 (1.61)
**Mediastinitis**	41 (0.79)
**Sepsis**	176 (3.37)
**Pneumonia**	495 (9.48)
**Wound infection**	488 (9.35)
**Stroke**	76 (1.46)

### SPScore model for simulated quality improvement and safety experiments

**Fig 1:**
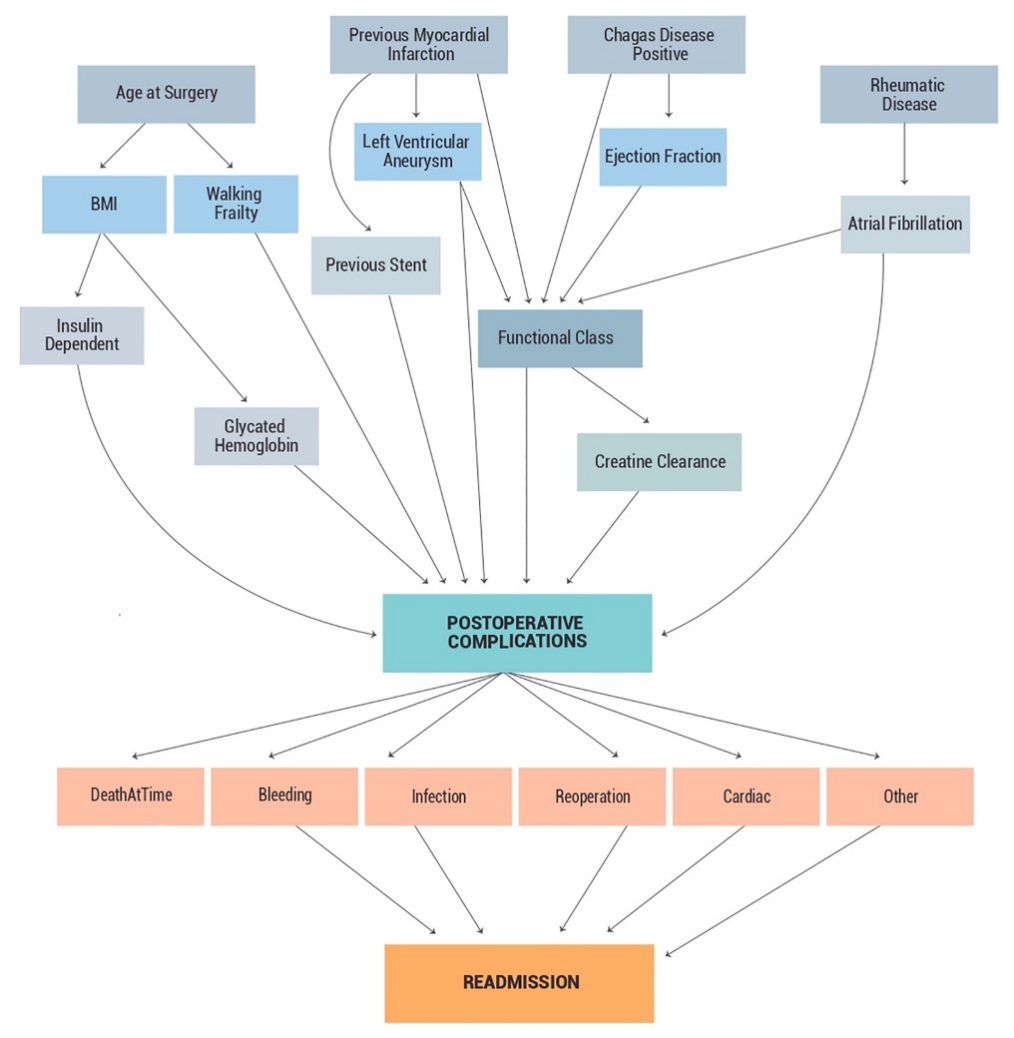
Machine learning model with an interrelated set of potential causes for all of the postoperative complications. A machine learning model were applied in our database with an interrelated set of potential causes for postoperative complications, as shown. Bleeding, infection, reoperation, cardiac complications and/or other complication were associated with readmissions.

As our registry provides data that directly reflects the daily practice of each of the participating institutions, it was also important to provide a mechanism that would allow each center to simulate potential Quality Improvement and Safety interventions. Thus, we generated a machine learning model with an interrelated set of potential causes for all postoperative complications (Fig 1).

**Fig 2:**
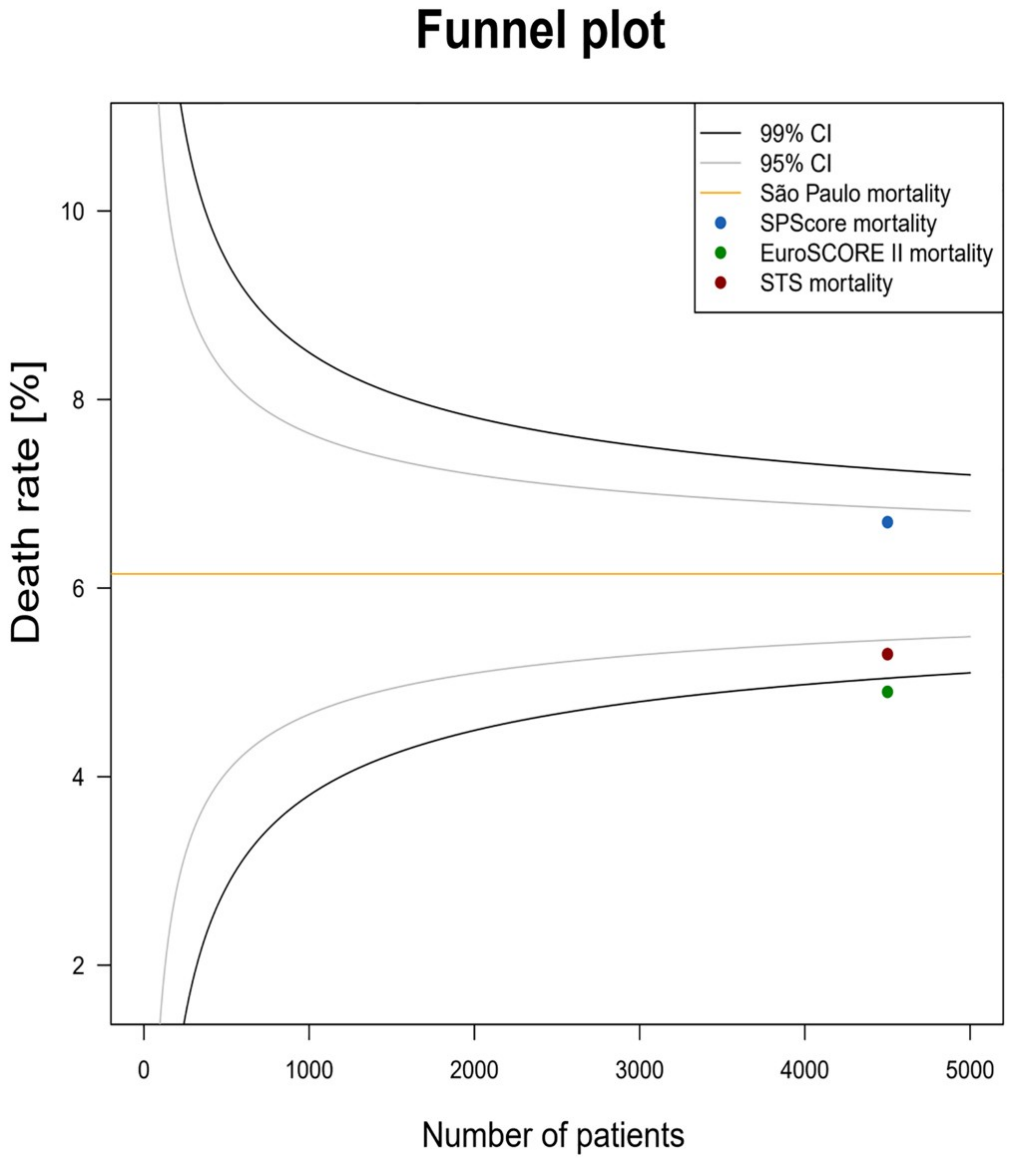
Funnel plot. The present funnel plot graph compares the predicted mortality of the risk score models evaluated in this study (represented in blue, SPScore; green, EuroSCORE II; and in red, STS). It is possible to observe that the SPScore was more accurate to predict the mortality of the evaluated sample.

Fig 2 shows a funnel plot comparing SPScore model with the EuroSCORE II and STS models in relation to the prediction of the overall mortality rate, and then compares it with the rate of this outcome for the state of São Paulo (administrative cardiovascular surgery database). We found that SPScore provides estimates that are closer to the actual values for the state. We suggest caution when interpreting the funnel plot, since the SPScore includes data from multiple comparators.

**Fig 3:**
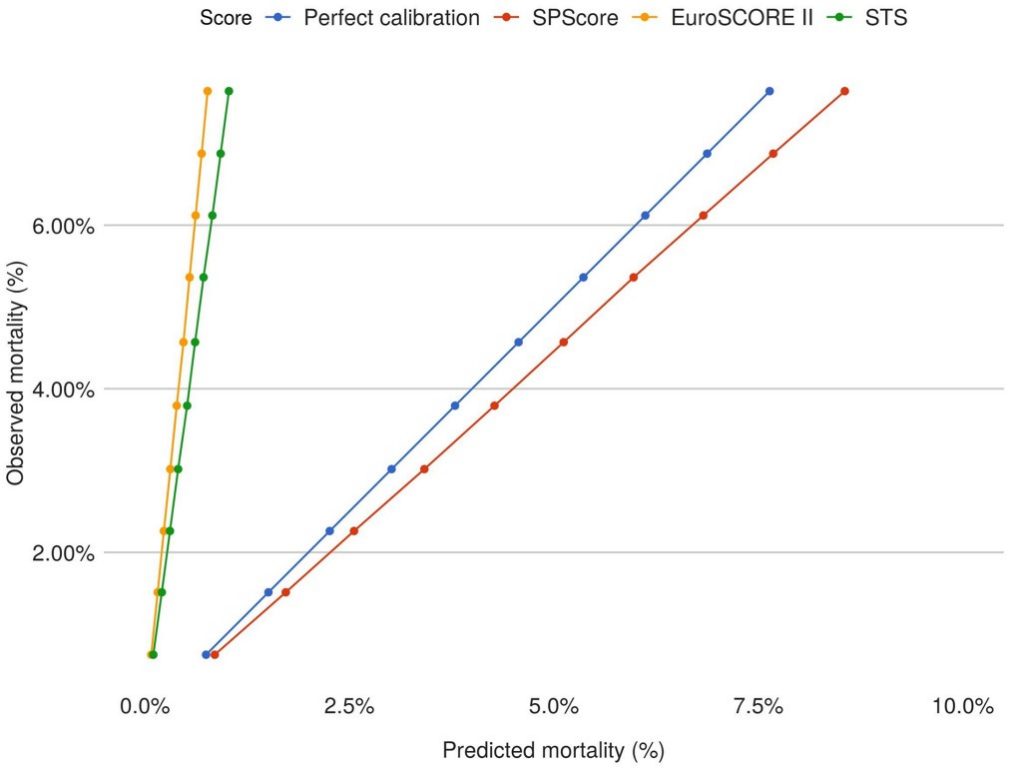
Comparative calibration curves for predicted and observed mortality in the SPScore, EuroSCORE II, and STS. This graph compares the predicted and observed mortality of the risk scores assessed in this study. The perfect state of the calibration is shown in blue; SPScore is represented in red, EuroSCORE II in orange, and finally, STS in green.

The calibration curves for SPScore, STS, and EuroSCORE II are presented in Fig 3. All scores demonstrated a relatively linear relationship between predicted and observed mortality, with SPScore demonstrating the best calibration statistics compared to EuroSCORE II and STS.

**Fig 4:**
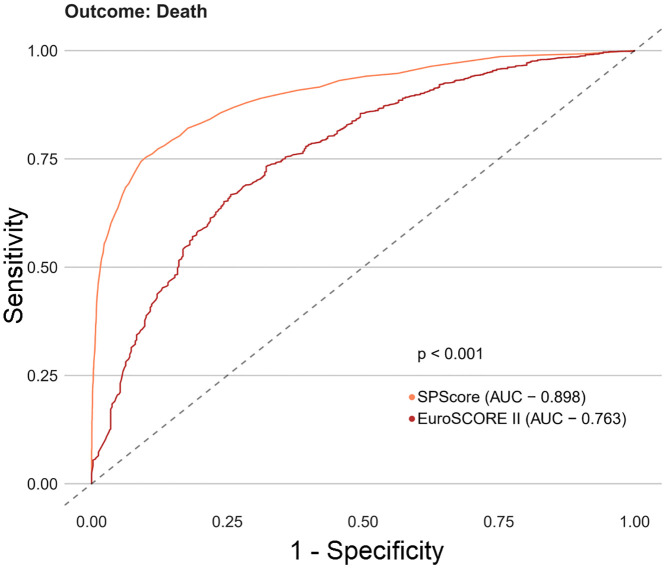
ROC curves for SPScore versus EuroSCORE II outcome death. In this figure, it can be seen that SPScore (AUC 0.898) is more accurate to predict deaths in REPLICCAR patients when compared to EuroSCORE II.

**Fig 5:**
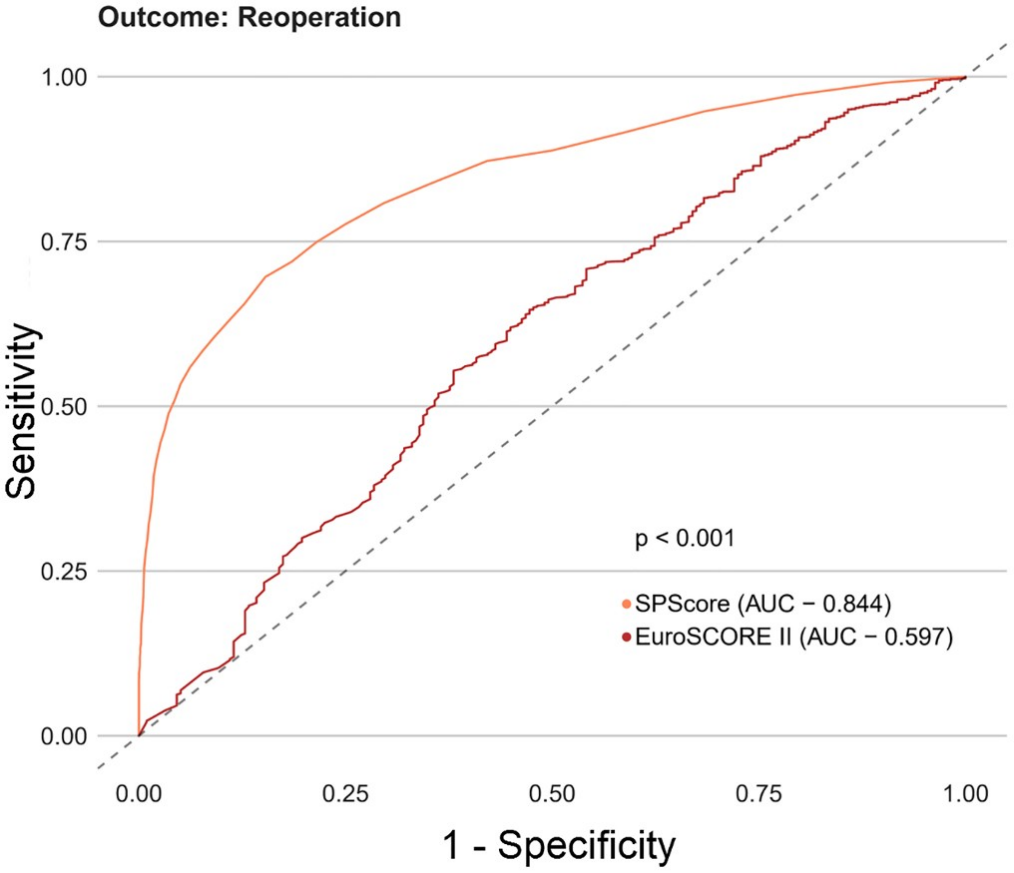
ROC curves for SPScore versus EuroSCORE II outcome reoperation. In this figure, it can be seen that SPScore (AUC 0.844) is more accurate to predict reoperation in REPLICCAR patients when compared to EuroSCORE II.

**Fig 6:**
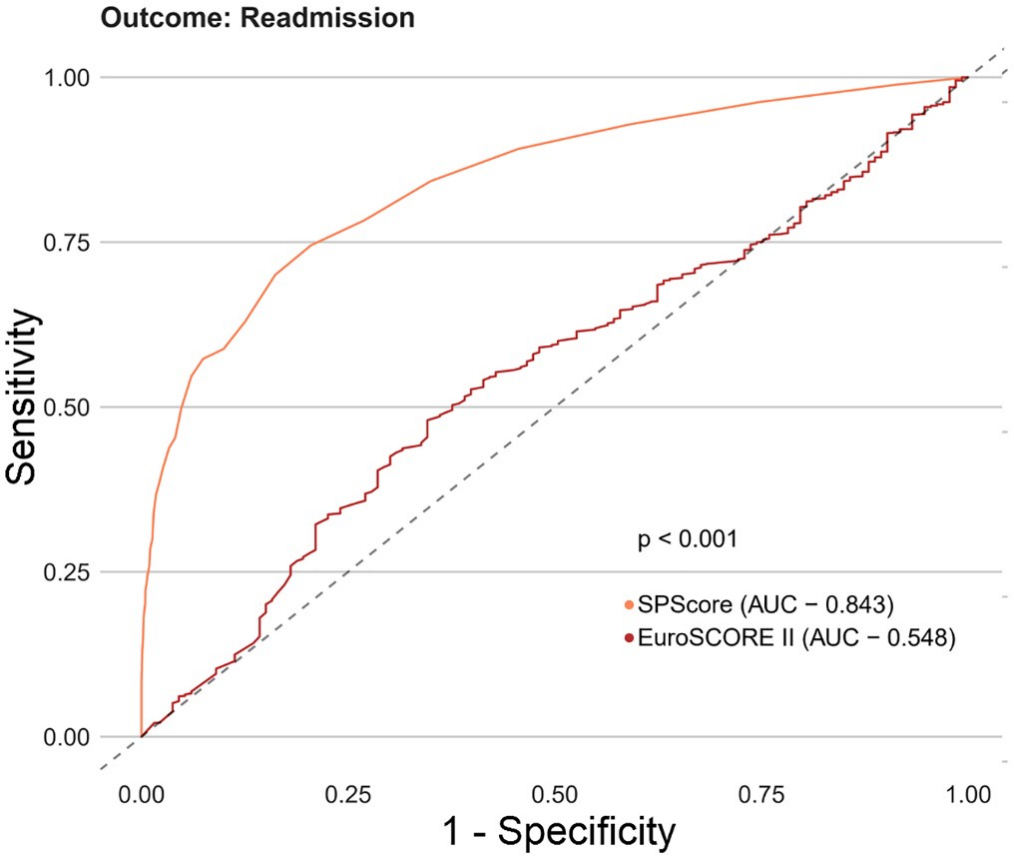
ROC curves for SPScore versus EuroSCORE II outcome readmission. In this figure, it can be seen that SPSCORE (AUC 0.843) is more accurate to predict readmission in REPLICCAR patients when compared to EuroSCORE II.

**Fig 7:**
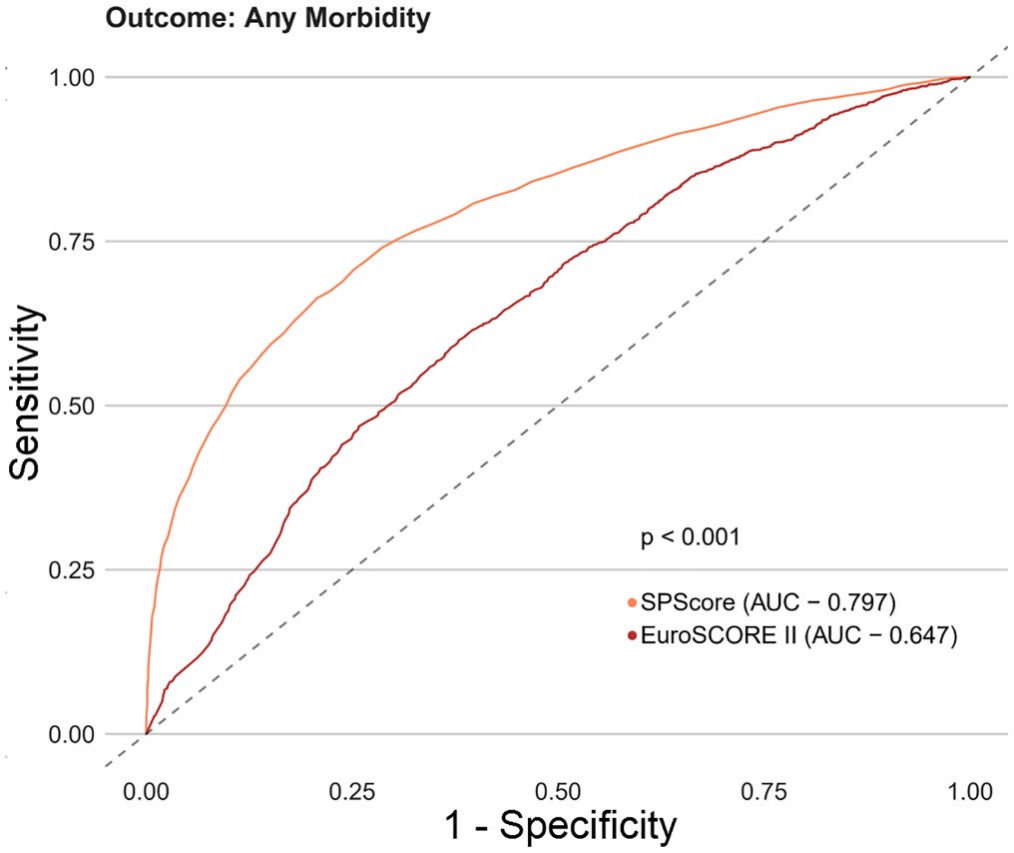
ROC curves for SPScore versus EuroSCORE II outcome any morbidity. In this figure, it can be seen that SPScore (AUC 0.797) is more accurate to predict any morbidity in REPLICCAR patients when compared to EuroSCORE II.

**Fig 8:**
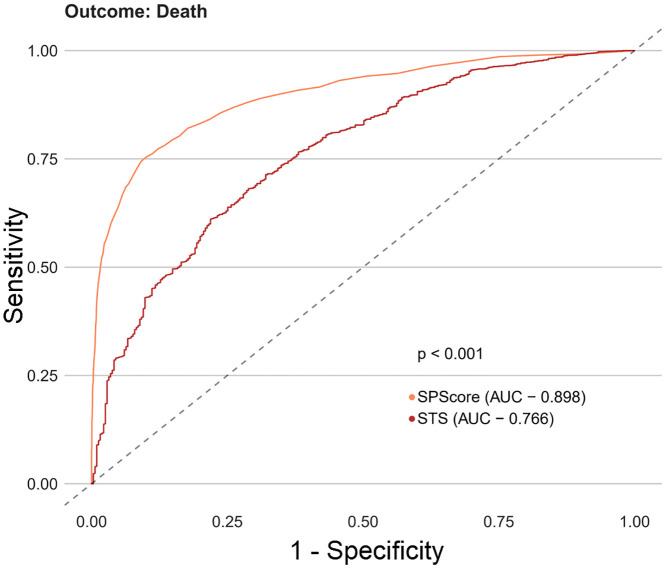
ROC curves for SPScore versus STS outcome death. In this figure, it can be seen that SPScore (AUC 0.898) is more accurate to predict deaths in REPLICCAR patients when compared to STS.

**Fig 9:**
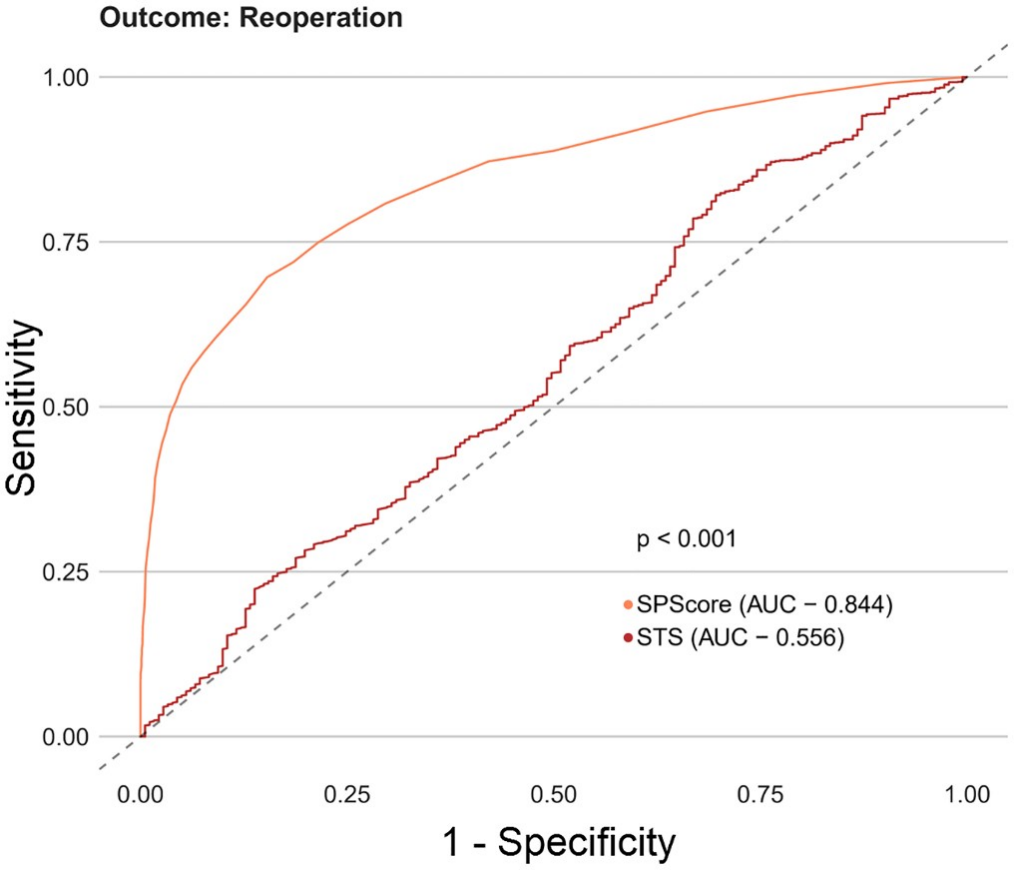
ROC curves for SPScore versus STS outcome reoperation. In this figure, it can be seen that SPScore (AUC 0.844) is more accurate to predict reoperation in REPLICCAR patients when compared to STS.

**Fig 10:**
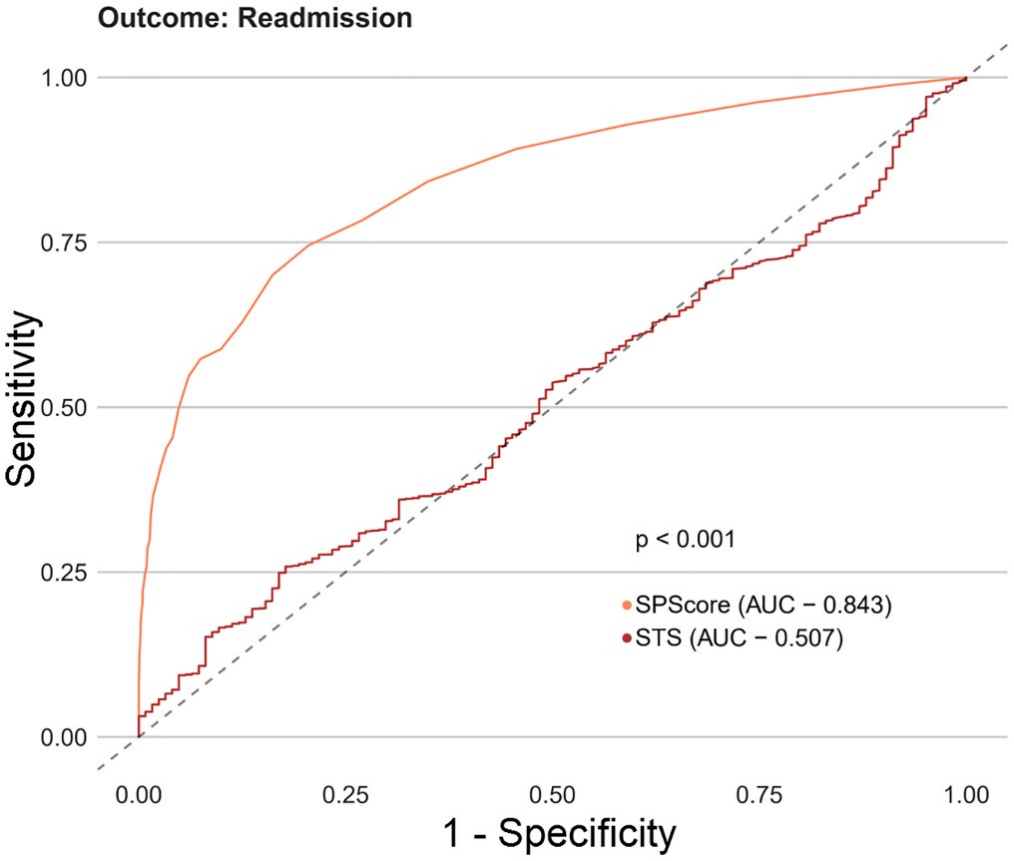
ROC curves for SPScore versus STS outcome readmission. In this figure, it can be seen that SPScore (AUC 0.843) is more accurate to predict readmission in REPLICCAR patients when compared to STS.

**Fig 11:**
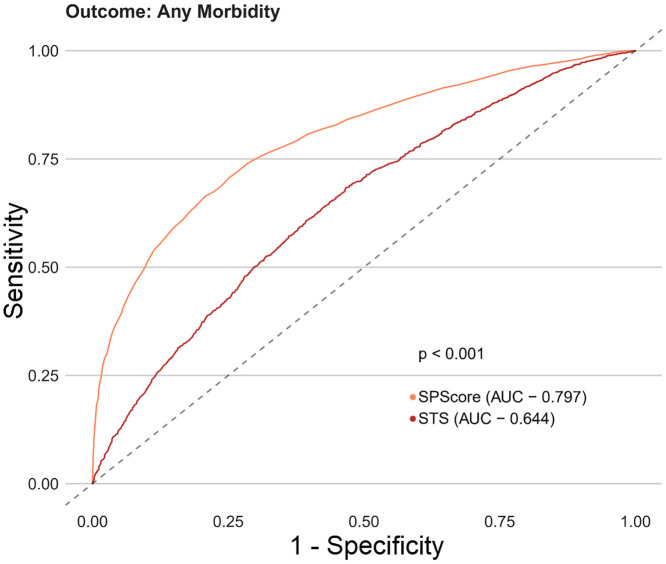
ROC curves for SPScore versus STS outcome any morbidity. In this figure, it can be seen that SPScore (AUC 0.797) is more accurate to predict any morbidity in REPLICCAR patients when compared to STS.

Among the SPScore models, the best performing model was the Random Forest. When evaluating the comparative prediction performance of the SPScore model in relation to the EuroSCORE II model, we found that SPScore outperformed EuroSCORE II in the prediction of mortality with an area under the curve of 0.90 for the SPScore model versus 0.76 for EuroSCORE II, reoperation (0.84 vs. 0.60), readmission (0.84 vs. 0.55), and any morbidity (0.80 vs. 0.65). All SPScore model outcomes were significantly greater than EuroSCORE II, with *p* values < 0.001 (Figs 4–7). We also evaluated the predictive performance of SPScore model versus STS, and we found that SPScore performed better than the STS, with an area under the curve significantly higher than those for STS for all study outcomes, with *p* values < 0.001 (Figs 8–11).

## Discussion

To the best of our knowledge, this is the first report of a state-wide Quality Improvement and Safety registry in cardiac surgery created in a developing country. We have outlined its main data collection infrastructure, along with a graphical interface for data exploration. In addition, we have presented SPScore, a machine learning model in which simulated Data Quality and Safety initiatives can be conducted, allowing prioritization over future interventions.

Initial efforts in Quality Improvement and Safety are traditionally attributed to Ernest Codman, using concepts borrowed from the technology industry [15]. As a consequence, patient outcomes have improved [16], particularly in contexts involving complex high-level procedures. Among the main initiatives of the Joint Commission’s was the 1986 project that led to the public dissemination of hospital data [17]. This path is promising, as continuous data-driven monitoring and feedback is a central tenet in quality improvement. However, facing these challenges is certainly worth our effort given the significant positive impact this program will have on our patients.

Cardiac surgery procedures in developing countries have been consistently reported to have higher mortality rates than those in developed countries [18]. For instance, Brazil alone recorded 9211 deaths (8.0%) from 115021 cardiac surgery cases performed between 2000 and 2003. In addition, other studies report that high mortality rates resulting from cardiac surgery are influenced by factors other than socioeconomic status [19–21]. These risk factors include the type of health care funding and the management of hospital centers [19, 20], initial level of illness severity, female gender [21], readmissions [22], and clinical and preoperative quality of life profiles [19]. It is interesting to note that even an increase in the volume of the procedure is not necessarily associated with an increase in patient quality of care [23], thus pointing to a complex causal network that, ultimately, leads to suboptimal clinical outcomes, with one of the most important factors related to the case mix. However, the case mix is not taken into account while defining quality policies and guidelines, because developing countries often rely on data and the corresponding evidence generated in developed countries, rather than collecting their own data. Clearly model-derived risks validated in one location or under certain conditions usually have lower performance when applied in another location and even in the same location over time. Another study developed the “RheSCORE model”, optimizing the prediction of mortality risk among patients undergoing valve surgery secondary to rheumatic valve disease, which outperformed the previously existing traditional scores with improved predictive performance [24].

Despite these issues, some studies have demonstrated that cardiac surgery outcomes in developing countries can be reduced over time through systematic team interventions [18, 25]. These interventions are particularly effective in relation to a systems-based approach, standardization, team building, consistent and accurate communication, and active management of changes and quality [26].

We evaluated the SPScore, EuroSCORE II, and the STS models as tools for risk prediction in cardiac surgery procedures. In our study, the SPScore outperformed EuroSCORE II and STS in predicting mortality, readmissions, reoperations, and any morbidity among Brazilian patients. Due to different population mixes and risk factors, there are significant differences in the prevalence of conditions and types of procedures between our study sample, EuroSCORE II, and the STS population. For example, rheumatic heart disease is a frequent condition in Brazil, with a prevalence of 14.5%. In contrast, in most European and North American countries the most frequent condition is degenerative heart disease. Therefore, it was actually expected that, given these differences, EuroSCORE II and STS would result in a poor calibration and discriminative power for the Brazilian population. We also found that, depending on the selected statistical model, EuroSCORE II can both overpredict or underpredict mortality among Brazilian patients. Even when overpredicting mortality, SPScore demonstrated a better calibration curve than the EuroSCORE II. Our findings suggest that the application of the REPLICCAR multicenter database and the creation of the SPScore model could help the quality improvement efforts to enhance surgical procedures.

Despite adding an important component to the Quality Improvement literature, our study does have limitations. First, given that our hospital network is geographically distributed, it is difficult to ensure reliability of the coding pattern of our procedures and outcomes. This limitation is currently being addressed through a measurement program related to inter-observer reliability followed by extensive site training, ultimately improving the consistency across our coders. Second, we opted for not including self-reported measures of quality of life or dysfunction. Although these measures constitute a critical piece in an assessment aimed at obtaining patient perspectives, it also increases the time required to evaluate individual patients. Third, despite our best efforts in controlling for missing rates, some of our variables had particularly high rates, specifically walking speed as a proxy for frailty. To control this limitation, we made use of imputation algorithms followed by sensitivity analyses to ensure that our conclusions were valid under different assumptions. Finally, given that our sample was not randomly drawn from a larger patient population, its external validity can be questioned. This limitation is currently being addressed through the inclusion of additional sites to the REPLICCAR registry, potentially reaching a third of all surgical procedures in the State of São Paulo. Finally, although social determinants of health have a significant effect on hospital readmissions and mortality [27], our dataset does not include these variables and therefore cannot be included in our models. Socioeconomic resources, such as lack of access to transportation and social support, affect patients’ ability to adhere to hospital discharge recommendations, leading to higher readmission risks [28]. This limitation might explain why our models demonstrate low variability in relation to clinical variables.

## Conclusion

We have described the internal structure of REPLICCAR, a novel registry for cardiac procedures in Brazil, with the aim of providing a model for similar centers in other developing countries. A regional risk assessment model, the SPScore, provided more precise estimates of death, readmission, reoperation, and any morbidity compared to EuroSCORE II and STS. A specific emphasis was placed on establishing mechanisms that enable scientific peers, health care providers, policymakers, and the general public to not only explore our data, but also to virtually simulate predictions to guide the choice of future quality improvement and safety interventions.

## Abbreviations

AVRR: Aortic Valve Replacement or Repair
BMI: Body mass index
CABG: Coronary artery bypass surgery
CI: Confidence intervals
EuroSCORE: European System for Cardiac Operative Risk Evaluation
FAPESP: São Paulo Research Foundation
HTA-NATSs/SES-SP: São Paulo Health Technology Assessment Network
MVRR: Mitral Valve Replacement or Repair
NYHA functional class: New York Heart Association functional class
PPSUS: Research Program for the Unified Health System
QI: Quality improvement
REPLICCAR: The São Paulo Cardiovascular Surgery Registry
RheSCORE: Rheumatic heart valve surgery SCORE
SMOTE: Synthetic Minority Over-sampling Technique
SPScore: São Paulo risk score
STS: Society of Thoracic Surgeons risk score
SUS: Unified Health System
